# Bivalent mRNA vaccine effectiveness against SARS-CoV-2 variants of concern

**DOI:** 10.1186/s12929-023-00936-0

**Published:** 2023-06-28

**Authors:** Monika Kumari, Shih-Chieh Su, Kang-Hao Liang, Hsiu-Ting Lin, Yu-Feng Lu, Kai-Chi Chen, Wan-Yu Chen, Han-Chung Wu

**Affiliations:** 1 Institute of Cellular and Organismic Biology, Academia Sinica, No. 128, Academia Road, Section 2, Nankang, Taipei, 11529 Taiwan; 2 Biomedical Translation Research Center (BioTReC), Academia Sinica, Taipei, 11529 Taiwan

**Keywords:** SARS-CoV-2, Variants of concern (VOCs), Bivalent mRNA vaccines, Vaccine efficacy

## Abstract

**Background:**

Sequential infections with SARS-CoV-2 variants such as Alpha, Delta, Omicron and its sublineages may cause high morbidity, so it is necessary to develop vaccines that can protect against both wild-type (WT) virus and its variants. Mutations in SARS-CoV-2’s spike protein can easily alter viral transmission and vaccination effectiveness.

**Methods:**

In this study, we designed full-length spike mRNAs for WT, Alpha, Delta, and BA.5 variants and integrated each into monovalent or bivalent mRNA-lipid nanoparticle vaccines. A pseudovirus neutralization assay was conducted on immunized mouse sera in order to examine the neutralizing potential of each vaccine.

**Results:**

Monovalent mRNA vaccines were only effective against the same type of virus. Interestingly, monovalent BA.5 vaccination could neutralize BF.7 and BQ.1.1. Moreover, WT, Alpha, Delta, BA.5, and BF.7 pseudoviruses were broadly neutralized by bivalent mRNA vaccinations, such as BA.5 + WT, BA.5 + Alpha, and BA.5 + Delta. In particular, BA.5 + WT exhibited high neutralization against most variants of concern (VOCs) in a pseudovirus neutralization assay.

**Conclusions:**

Our results show that combining two mRNA sequences may be an effective way to develop a broadly protective SARS-CoV-2 vaccine against a wide range of variant types. Importantly, we provide the optimal combination regimen and propose a strategy that may prove useful in combating future VOCs.

**Supplementary Information:**

The online version contains supplementary material available at 10.1186/s12929-023-00936-0.

## Background

Throughout the COVID-19 pandemic, researchers in academic laboratories and pharmaceutical companies around the world have worked tirelessly to resolve the devastating situation. Only a few months after the release of the severe acute respiratory syndrome coronavirus 2 (SARS-CoV-2) sequence, the first COVID-19 vaccines entered the clinical trial phase [1]. These vaccines were deemed a great success, as infection and mortality rates were greatly reduced with their deployment [2, 3]. In spite of this progress, there is still no fully effective vaccine or cure for the virus that has been produced. In fact, one individual who received two doses of COVID-19 vaccines even contracted an infection that warranted further booster shots [4]. Due to its inevitable evolution, the SARS-CoV-2 sequence continues to cause unprecedented health problems around the world. Most mutations arise with little consequence and do not significantly affect the efficacy of current vaccines. However, a few variants carry immune-escape mutations, which reduce the efficacy of existing vaccines [5].

Mutations in the spike (S) protein may confer advantages to the virus in terms of increased transmissibility potential and immune evasion. As such, several variants with S protein mutations were designated as VOCs in late 2020, and these variants have largely been responsible for most epidemic waves around the world. The major VOCs are Alpha, Beta, Delta, and Omicron [6, 7], and for each of these strains, mutations in the S protein have allowed the virus to escape neutralizing antibody responses and infect vaccinated individuals [8]. Existing COVID-19 vaccines mainly target the S protein sequence of WT SARS-CoV-2 because it is reasonably well conserved and a major target for neutralizing antibodies [5]. However, Omicron BA.1 carries many mutations in its S protein, so it can successfully escape antibody binding in vaccinated individuals and has become one of the most infectious VOCs [9–11]. Moreover, mutations to the BA.1 have given rise to new Omicron sublineages with similar or enhanced escape properties.

Currently, BA.5 has displaced most of the other Omicron sublineages to become the dominant strain in many countries around the world. BA.5 was first identified in South Africa, and within only a few months, the lineage emerged in the USA and became dominant, eventually accounting for nearly 80% of all infections [11, 12]. Certain key alterations in BA.2 S glycoprotein, specifically L452R and F486V, later occurred in the BA.4 and BA.5 sublineages. These modifications may allow the virus to enter host cells more efficiently and evade neutralizing antibodies induced by the WT COVID-19 vaccines [13]. GISAID reported that BA.5 and its descendent lineages accounted for 68.1% of all sequences submitted from 2 to 8 January 2023 [14]. Recently developed triple vaccines from AstraZeneca (AZD1222) and Pfizer (BNT162b2) have low neutralization titers against BA.4/5 when compared with BA.1 and BA.2. AZD1222 induces a neutralization titer toward BA.4/5 that is approximately 1.8-fold lower than that toward BA.2, while BNT162b2 induces a neutralization titer for BA.4/5 that is approximately 3.1-fold lower than that for BA.2 [15]. As would be expected, clinical vaccine efficacies against emergent Omicron sublineages have been greatly reduced due to the poor induction of neutralizing titers [16, 17].

Recently, the BA.5 sublineages called BF.7 and BQ.1.1 have rapidly spread in many regions and become dominant variants [18]. BF.7 and BQ.1.1 both carry the R346T mutation on a BA.5 backbone. In addition, BQ.1.1 carries K444T and N460K mutations, which are associated with its increased prevalence. According to a recent literature survey, bivalent booster shots may increase the neutralizing antibody titer as compared with monovalent vaccines. However, the bivalent booster shots still induce low neutralizing antibody titers for BF.7 and BQ1.1, which are respectively 1.5- and 7.2-fold lower than that for BA.5 [19]. It has been documented that most vaccines cannot neutralize the BQ1.1 subvariant [20]. Hence, the introduction of mutations in the S protein not only enhance neutralization escape but also reduce vaccine efficacy. To date, no clinical strategy has been developed to compensate for low vaccine efficacy against new VOCs.

In light of the poor protection provided by current vaccines from BA.5 Omicron sublineages, we investigated whether combinations of mRNAs encoding different variants might better protect against recent or future VOCs. Here, we found that multivalent mRNA-lipid nanoparticle (LNP) formulations generally induced higher neutralizing antibody titers against a broad selection of VOCs as compared with monovalent mRNA-LNP formulations. In particular, we evaluated the breath of neutralizing activity in immunized mouse sera against Alpha, Beta, Delta, BA.5 BF.7, and BQ.1.1. Combinations of BA.5 mRNA with Alpha, Beta, or Delta mRNA showed strong cross-neutralizing antibody responses against relevant VOCs.

## Methods

### Production of modified mRNAs by in vitro transcription (IVT)

The WT, Alpha, Delta, and BA.5 SARS-CoV-2 S cDNA plasmids were kindly provided by Dr. Yu‑Chi Chou, National RNAi Core Facility, Academia Sinica, Taiwan. In brief, synthesized DNA fragments of SARS-CoV-2 genes encoding S protein were purchased from Integrated DNA Technologies and subcloned into the KpnI and EcoRI sites of pcDNA3.1 (+) expression plasmid using a GenBuilder Cloning Kit (GenScript).

The DNA templates were kindly provided by Dr. Mi-Hua Tao's lab, Academia Sinica. Briefly, DNA templates were constructed to contain a T7 promoter site, a codon-optimized various SARS-CoV-2 S protein (WT, Alpha, Delta, and BA.5), a 5’ UTR, IgG kappa leader sequence, a poly(A) tail region, and the alpha-globin gene 3’ UTR. Prior to IVT, the plasmid was linearized using EcoRV and purified with the NucleoSpin Gel and PCR Clean-up Kit (Macherey & Nagel Co. Düren, Germany). mRNA was synthesized according to the manufacturer’s recommendations using HiScribe T7 (NEB, MA, USA) with co-transcriptional CleanCap® AG (Trilink, CA, USA) and N1-methyl-pseudouridine (Trilink, CA, USA). Synthesized mRNA was purified by DNase I (NEB, MA, USA) digestion followed by LiCl (Invitrogen, Thermo Fisher Scientific, Waltham, MA, USA) precipitation and a 70% ethanol wash. Cellulose-based purification was performed to remove dsRNA from the transcribed mRNA. The final product was stored at − 80 °C.

### Preparation of mRNA-LNPs

LNP formulations were prepared using a previously described method [21]. Briefly, four types of lipids were solubilized in ethanol: SM-102 (MedChemExpress, NJ, USA), DSPC (Avanti Polar Lipids, NY, USA), cholesterol (Sigma, MA, USA) and DMG-PEG 2000 (MedChemExpress, NJ, USA). The lipids were then mixed with a molar ratio of 50:10:38.5:1.5. The lipid mixture was combined with an aqueous sodium acetate buffer (25 mM, pH 4.5) containing mRNA at a flow rate ratio of 1:3 using NanoAssemblr® IGNITE NxGen Cartridges (Precision NanoSystems Inc., BC, Canada). LNP-encapsulated mRNA samples were dialyzed against PBS (pH 7.4) at 4 °C. Then, the mRNA-LNPs were concentrated using Amicon Ultra Centrifugal Filters (10 K MWCO; Millipore, Burlington, MA, USA) and passed through a 0.45-mm filter.

### Characterization of mRNA-LNP

The particle size distribution, polydispersity index (PDI) value, and zeta potential of each SARS-CoV-2 S protein mRNA-LNP were analyzed by dynamic light scattering (DLS, Zetasizer Nano ZS, Malvern Instruments, UK). The sample was diluted 100-fold and equilibrated for 120 s at 25 °C prior to size and zeta potential measurements. The hydrodynamic diameter (z-average) and zeta potential of mRNA-LNP were analyzed by Zetasizer software, version 7.11 (www.malvern.com). The morphology of mRNA-LNP was observed in a dry state using cryogenic transmission electron microscopy (cryo-TEM, Tecnai F20, Philips, Eindhoven, the Netherlands). Briefly, the sample solution was diluted 10-fold and transferred onto a 300-mesh copper grid covered with porous carbon film (HC300-Cu, PELCO) before blotting and plunging in a 100% humidity temperature-controlled chamber by Vitroblot (FEI). The copper grids were stored under liquid nitrogen and transferred to the electron microscope on a cryo-stage for imaging. The mRNA encapsulation efficiency (EE%) and the concentration were determined by using the Quant-iT RiboGreen RNA assay kit (Invitrogen, Thermo Fisher Scientific, Waltham, MA, USA). The mRNA integrity of free mRNA and mRNA-LNP was analyzed by an agarose gel retardation assay. mRNA-LNP complexes were solubilized with 1% Triton X-100, and the integrity of released mRNA was inspected on the agarose gel.

### Evaluation of in vitro SARS-CoV-2 S protein expression by flow cytometry

SARS-CoV-2 S protein mRNA-LNPs (WT, Alpha, Delta, and BA.5) were individually transfected into HEK293T cells and cultured at 37 °C in DMEM medium containing 10% FBS for 24 h. Then, the cells were collected and centrifuged. The cell pellet was washed with PBS via centrifugation, followed by incubation with fixation and permeabilization solution (BD, catalog no. 554,714) for 20 min at 4 °C. Cells were then stained with 1 µg/ml RBD domain of S protein-specific monoclonal antibodies (K-RBD-mAb-75) for 1 h at 4 °C, followed by incubation with PE-conjugated goat anti-mouse IgG (1:500) for 1 h at 4 °C. The total S protein levels were determined from both permebilized and non-permebilized cells. The fluorescent signals were detected by flow cytometer; a minimum of 1 × 10^4^ events were recorded for each sample and analyzed with FlowJo software.

### Mouse immunization

All procedures involving animal studies were approved and performed in accordance with guidelines set by the Institutional Animal Care and Use Committee (IACUC) at Academia Sinica, Taiwan. Groups of 6- to 8-week-old BALB/c mice were immunized via intramuscular injection with 10 µg of indicated SARS-CoV-2 S protein mRNA-LNP or control solution (saline) at weeks 0, 2, and 4. Serum samples were collected 6 weeks after the first immunization and stored at − 80 °C until further use.

### Analysis of binding affinity of anti-SARS-COV-2 antibodies from immunized mouse sera

Measurements of binding antibody levels to various SARS-CoV-2 strains were performed on sera from immunized mice, as previously described [22]. Briefly, ELISA plates were coated with 0.5 µg/ml of S protein purchased from ACRO Biosystems (Alpha strain, SPN-C52H6; Delta strain, SPN-C52He; Omicron BA.5, SPN-C522e) and incubated at 4 °C overnight, followed by blocking with PBS containing 1% bovine serum albumin (BSA) at RT for 2 h. After blocking, the wells were washed twice with PBS. Then, sera that were serially diluted with 1% BSA in PBS were added into each well in triplicate, and the plate was incubated at room temperature for 1 h. After an incubation period, the plates were washed three times with PBS containing 0.1% Tween-20 (PBST0.1) and then incubated for 1 h with peroxidase-affinipure goat anti-mouse IgG (H + L) (Jackson ImmunoResearch) (1:5000 dilution). After three washes with PBST0.1, signal was produced using 3,3’5,5’-Tetramethylbenzidine (TMB) substrate (TMBW-1000-01, SURMODICS). Finally, the reaction was stopped with 3 N HCl, and absorbance was measured at 450 nm by an ELISA reader (Versa Max Tunable Microplate Reader; Molecular Devices).

### Pseudovirus neutralization assay

Blood samples were collected from mice at six weeks after the first boost, and the sera were used to determine the neutralization activity against different SARS-CoV-2 pseudoviruses. The pseudovirus neutralization assays were performed using SARS-CoV-2 pseudotyped lentiviruses expressing full-length S protein and firefly luciferase in HEK293T cells that overexpressed human ACE2 (HEK293T/hACE2; purchased from the National RNAi Core Facility, Academia Sinica, Taiwan). The serum from each mouse was serially diluted using 1% FBS DMEM and pre-incubated with 1000 TU SARS-CoV-2 pseudovirus for 1 h at 37 °C. After incubation, the mixtures were added to 1 × 10^4^ HEK293T/hACE2 cells pre-seeded in each well of a 96-well white plate (SPL Life Science) for 24 h at 37 °C. The pseudovirus-containing culture medium was then replaced with 10% FBS DMEM for an additional 48-hour incubation. Next, ONE-Glo luciferase reagent (Promega) was added to each well for 3-minute incubation at 37 °C to measure firefly luciferase activity. Luminescence was measured using a microplate spectrophotometer (Molecular Devices) to determine pseudovirus infection efficacy. The half-maximal inhibitory concentration (IC_50_) was calculated by nonlinear regression using Prism software version 8.1.0 (GraphPad Software Inc.). The average IC_50_ value for each experimental group was determined from three independent experiments.

## Results

**Fig. 1:**
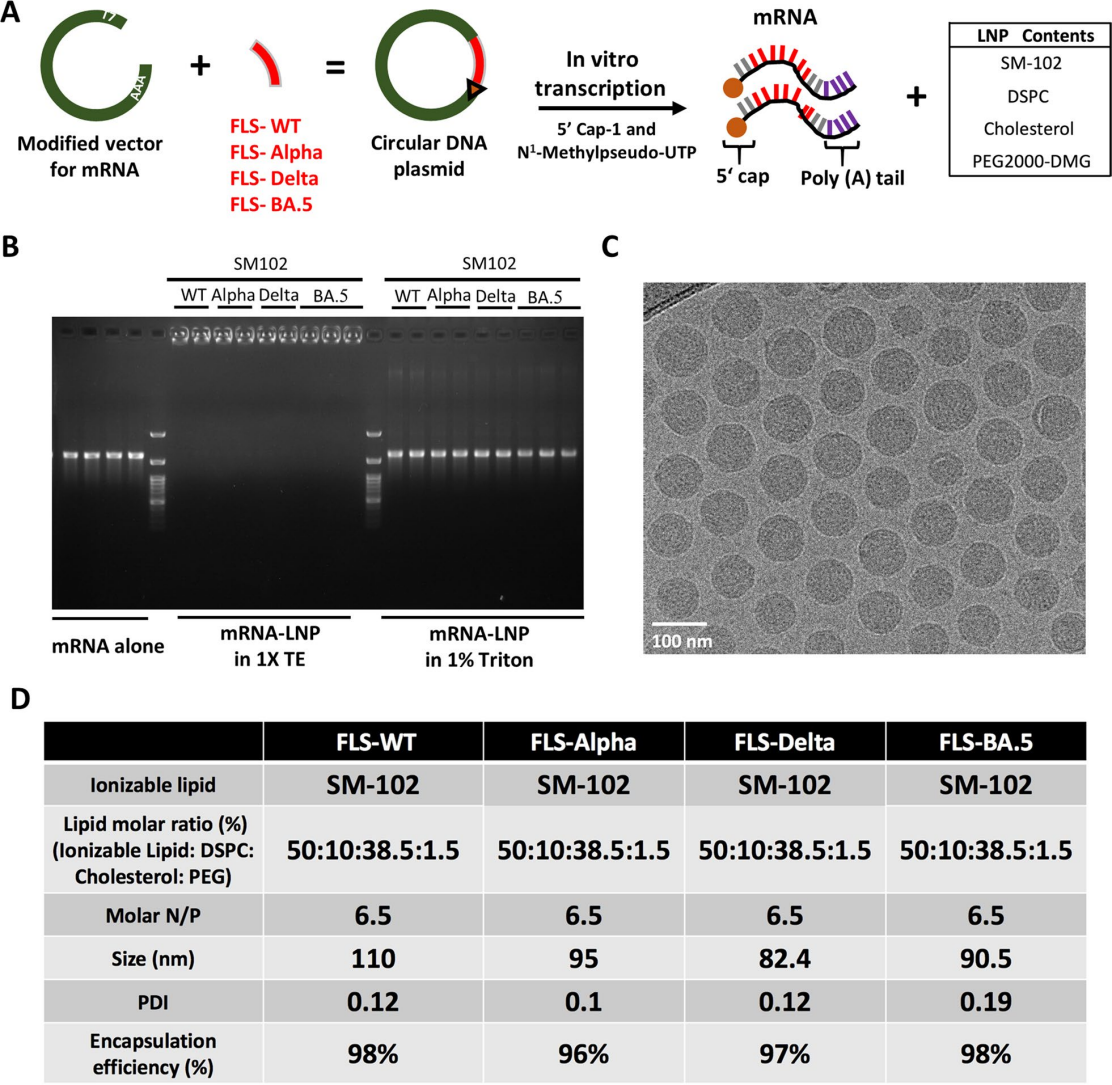
Characterization of mRNA and mRNA-LNPs. **A** Schematic diagram depicts the mRNA synthesis steps. **B** Gel electrophoresis assay. mRNA-LNPs were run in the 1X TE buffer. Naked mRNAs served as the negative control. **C** Cryo-TEM images illustrate the structure of mRNA-LNPs. **D** Summary table shows the molar ratio and physiochemical characterization of mRNA-LNPs

We generated SARS-CoV-2 mRNAs for S proteins from WT virus and its variants, including Alpha, Delta, and Omicron BA.5. Our selection of BA.5 was based on the fact that it possesses an identical S protein to that of BA.4 and is closely related to BA.2 [23], as well as the fact that its sublineages BF.7 and BQ1.1 are rapidly gaining global dominance. Furthermore, neutralizing antibodies against BA.4/5 show cross-neutralization of other Omicron sublineages [24]. In each mRNA sequence, the gene coding sequence was flanked by a 5’ and 3’ UTR region from the human hemoglobin subunit alpha 1 mRNA to regulate the mRNA stability and protein expression (Fig. 1A). The integrity of in vitro-synthesized mRNA was monitored with an agarose gel retardation assay (Fig. 1B). A NanoAssemblr® Ignite microfluidic mixing device was used to prepare mRNA-LNP complexes with respective mRNAs.

### Physiochemical characterization of mRNA-LNP complexes

The integrity of mRNAs packed into LNPs was characterized by agarose gel electrophoresis assays. The results in Fig. 1B show that mRNA-LNP complexes were trapped within the gel well when run with 1X TE buffer only. However, further treatment of mRNA-LNP complexes with 1% TritonX-100 generated a band in the same relative position as naked mRNAs. These data indicate that mRNA was successfully packed into LNPs, and disrupting the LNPs could release the mRNA without causing degradation and affecting mRNA integrity.

The average particle sizes of the mRNA-LNP complexes were measured at a range between 80 and 110 nm using Dynamic light scattering (DLS). Most importantly, the polydispersity index (PDI) values lower than 0.3 indicated uniform distribution and lack of aggregation of the mRNA-LNP complexes in an aqueous solution (Fig. 1D). Cryo-EM imaging of the mRNA-LNP complexes also revealed a homogenous distribution and spherical structure of each formulation (Fig. 1C).

### Validation of protein expression in vitro

**Fig. 2:**
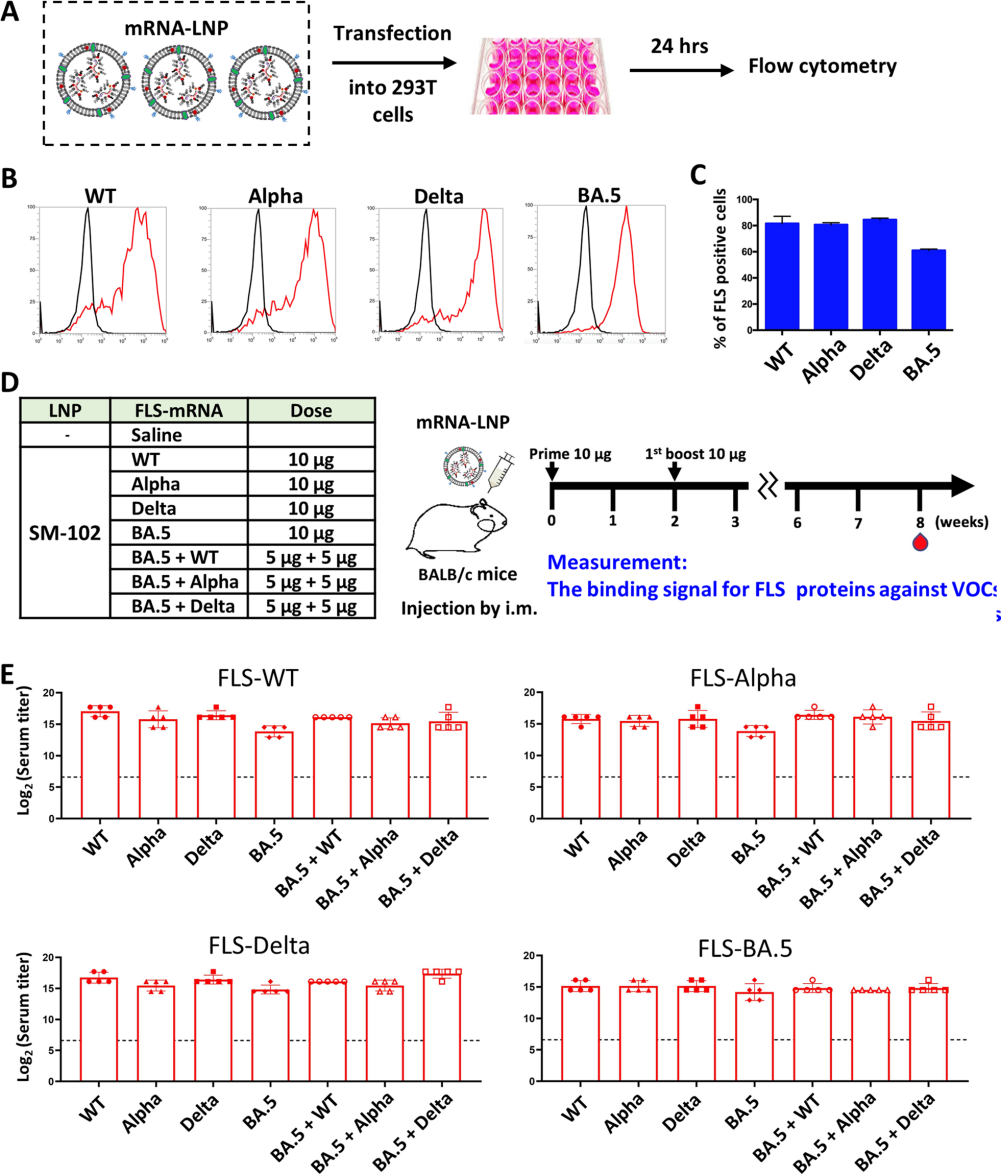
Transfected HEK293T cells with respective mRNAs were observed using flow cytometry. **A** Schematic diagram illustrates the transfection process. **B**, **C** Flow cytometry data showed the successful transfection of mRNAs. **D** Schematic diagram depicts the animal immunization process. **E** ELISA was used to evaluate the binding activity of immunized serum against proteins from each variant. All experiments were performed in triplicate; standard deviations are shown as error bars. Graphical data are shown as mean ± SEM. The WT treatment was used as a control group in a two-tailed Student’s t test; *p < 0.05, **p < 0.01

mRNA-LNP complexes containing one of four different types of mRNA construct (WT, Alpha, Delta or BA.5) were applied to HEK293T cells. The mRNA-LNP treatment scheme is shown in Fig. 2A. After an incubation period, cells were collected using dissociation buffer and analyzed by flow cytometry with an in-house-generated chimeric antibody called k-RBD-chAb-75. This antibody can bind to S protein of WT virus and its variants [25]. Each different mRNA could successfully induce S protein expression in the transfected cells. After binding of cells with the chimeric antibody, the shifted peak in the flow cytometry data was used to calculate the percentage of cells expressing full-length S protein (Fig. 2B C).

### Bivalent mRNA-LNP complexes induce robust antibody responses in mice

After confirming that each mRNA-LNP complex could successfully induce protein expression in the cell-based assay, the immunogenic effects were assessed in BALB/c mice. Eight groups of 6- to 8-week-old mice were immunized twice with intramuscular (i.m) injections of monovalent or bivalent mRNA-LNP complexes. Mice receiving saline served as the negative controls (Fig. 2D). To investigate the functionality of bivalent mRNA-LNPs, we chose to inject combinations of BA.5 + WT, BA.5 + Alpha, BA.5 + Delta. These combinations were compared with monovalent mRNA-LNPs. An ELISA-based method was performed to evaluate the binding of serum antibodies to WT, Alpha, Delta, and BA.5 recombinant S proteins. The data showed no significant difference between the binding activities of antibodies generated by monovalent and bivalent vaccines (Fig. 2E). Despite this lack of difference, the bivalent vaccine contains half the amount of mRNA for each targeting antigen compared to the corresponding monovalent vaccines. Nevertheless, the antibodies generated by monovalent and bivalent vaccines were similarly able to bind to the virus. Thus, the bivalent vaccine may not be substantially more effective than the monovalent vaccine in terms of antibody binding activity, and both vaccines are effective at preventing infection. Importantly, previous studies have reported a poor correlation between antibody binding activity and neutralizing activity toward SARS-CoV-2, suggesting that many antibodies produced after vaccination may bind to the S protein but fail to neutralize the virus [26]. Therefore, we further performed pseudovirus-neutralizing activity assays to analyze the potencies of antibodies generated via vaccination.

To track potential negative side effects, body weight was evaluated each week after immunization of mice with monovalent vaccines (WT or BA.5) or bivalent vaccine (WT + BA.5). The body weights decreased slightly after immunization with both types of vaccine, but the animals regained weight to achieve pre-injection levels after 7 days (Additional file 1: Fig. S1). Overall, these data indicate that immunization had no long-term negative effects on body weight. In line with these results, previous research has demonstrated that mice receiving LNP-mRNA vaccinations may experience a decrease in body weight for a few days after the vaccination; however, the body weights soon return to the original levels [27]. In another study, the authors compared the side effects of LNP-mRNA vaccination of BALB/c mice administered via two different routes, i.e., intravenous (i.v.) and intramuscular (i.m.). The data showed that i.v. administration induced rapid onset multifocal myopericarditis, but i.m. administration did not. Furthermore, the animal body weights decreased briefly after vaccination but returned to the initial values after 7 days [28]. Another recent study showed that the local and systemic reactions experienced following the administration of a bivalent booster dose are similar to those reported after monovalent booster doses [29]. Thus, our data are consistent with previously published findings and do not suggest that any additional adverse effects will be observed for bivalent vaccines as compared with monovalent vaccines.

### Bivalent mRNA vaccination induces cross-variant neutralization of SARS-CoV-2 variant pseudoviruses

**Fig. 3:**
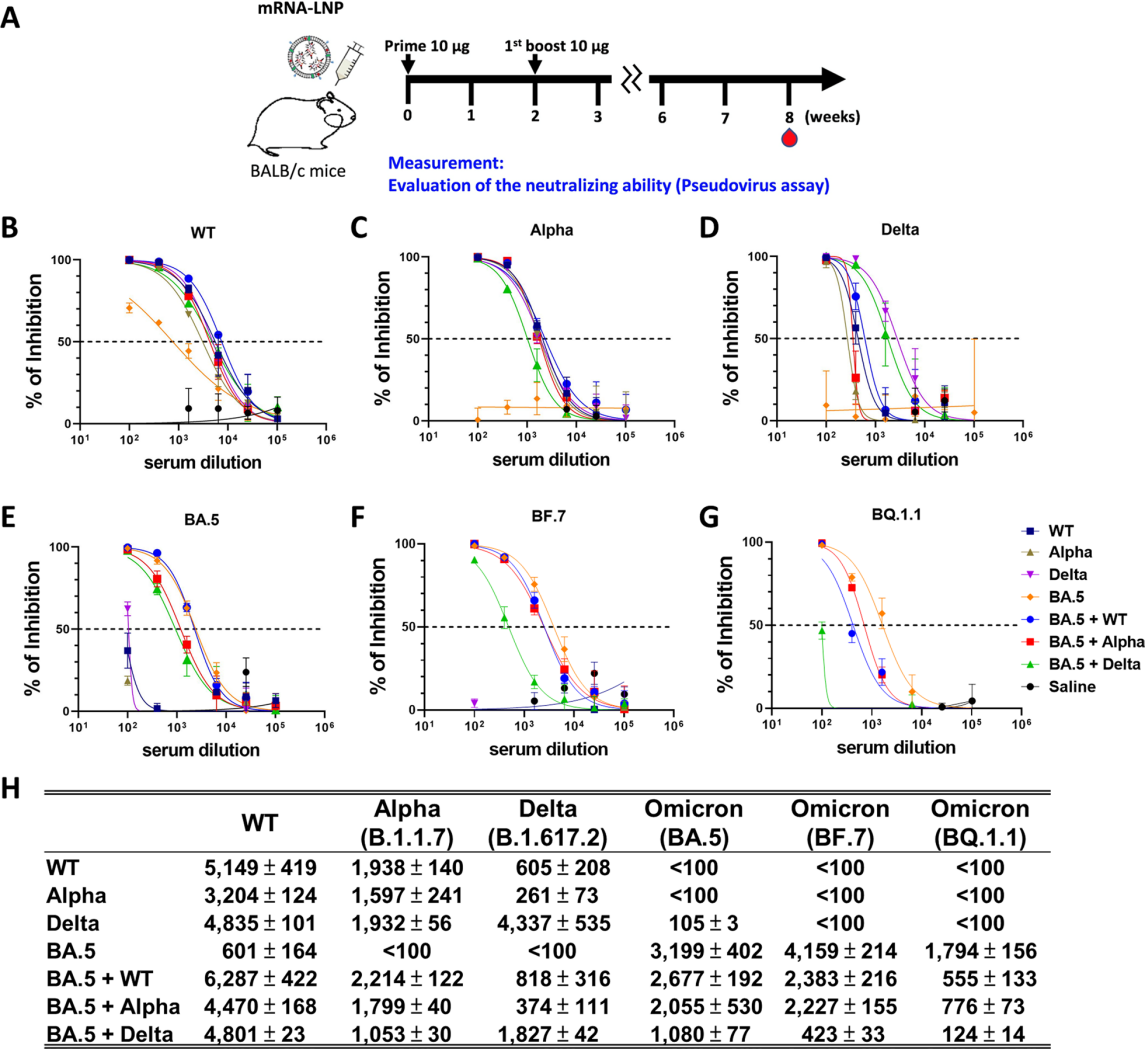
Bivalent mRNA vaccination induces neutralizing activity against SARS-CoV-2 variant pseudoviruses. **A** Mouse model immunization and blood sample collection schedules. **B**–**G** Pseudovirus neutralization assay on serum collected from mice receiving monovalent or bivalent mRNA vaccination against wild-type (WT), Alpha, Delta, BA.5, BF.7, and BQ.1.1 pseudoviruses. **H** Half-maximal inhibitory concentrations (IC_50_) values for serum of monovalent or bivalent mRNA-vaccinated mouse against SARS-CoV-2 variant pseudoviruses. All experiments were performed in triplicate; standard deviations are shown as error bars

Neutralizing activity was evaluated using sera from post-vaccinated animals, as depicted in Fig. 3A. In pseudovirus neutralization experiments, mice immunized with monovalent WT, Alpha, or Delta mRNA failed to neutralize the BA.5 pseudovirus. Similarly, sera from mice immunized with monovalent BA.5 mRNA were also incapable of neutralizing Alpha and Delta pseudoviruses, and the neutralization of WT pseudovirus was minimal (Fig. 3B–H and Additional file 2: Fig. S2). In contrast, sera from mice immunized with bivalent mRNA vaccines (BA.5 + WT, BA.5 + Alpha, or BA.5 + Delta) showed broad abilities to neutralize of WT, Alpha, Delta, and BA.5, BF.7, and BQ1.1 pseudoviruses. It was also found that bivalent mRNA vaccines induced significantly stronger neutralizing antibody titers against the BA.5 pseudovirus compared to monovalent WT, Alpha, or Delta vaccines (Fig. 3E and Additional file 2: Fig. S2). In comparison to other combination regimens, BA.5 + WT appeared to be the most effective. Still, the mice vaccinated with bivalent BA.5 + WT exhibited lower neutralizing antibody titers for BF.7 and BQ1.1 (1.1- and 4.8-times lower) than for BA.5. Notably, the sera from monovalent BA.5 vaccine recipients showed lower neutralizing resistance to BQ1.1 than did bivalent vaccine recipients, suggesting that BQ1.1 may have high neutralizing resistance as compared with other VOCs (Fig. 3H). It may therefore be possible to induce better serum neutralizing activity against a broad range of SARS-CoV-2 variants with the use of a bivalent mRNA vaccine containing S protein mRNAs from both previous and current variants.

## Discussion

Despite the availability of multiple COVID-19 vaccines, the continued emergence of new variants has diminished vaccine effectiveness. Previous studies have evaluated the effectiveness of repeated immunization for different viral variants, revealing that the approach can be beneficial due to affinity maturation of B cells. However, it is important to note that repeated boosting with vaccines against homologous variants may not be effective at preserving the epitope repertoire [30]. In only a short time, the Omicron sublineages have superseded the other variants, making it difficult to predict the efficacies of vaccines produced for earlier strains. It has been shown that homologous or heterologous booster doses of mRNA vaccines result in low (36- to 40-fold reduced) neutralizing ability against Omicron BA.1 [31]. In light of this problem, researchers must urgently fine-tune available vaccines, tailor booster doses, or produce variant-based antigens to ensure our ability to fight SARS-CoV-2 VOCs. Recently, studies have demonstrated that booster doses of mRNA-1273, BNT162b2, or Ad26.COV2 vaccines could help to neutralize the Omicron B.1.1.529 sublineage [32–34]. Boosting with bivalent vaccines is gaining attention due to its enhancement of neutralizing antibody levels, as well as its improved cross-reactivity toward multiple variants compared to monovalent vaccines [35, 36]. Furthermore, Fang et al. showed that a bivalent booster dose significantly outperformed a monovalent booster shot. The authors of that study found that a monovalent booster shot against WT, Delta, or BA.2 S proteins increased neutralizing antibody titers for BA.2.12.1, BA.2.75, and BA.2; however, the effects were minimal for BA.4/5. Nevertheless, they further showed that a bivalent booster dose of Delta and BA.2 antigens broadly neutralizes most Omicron sublineages, including BA.5 [37]. Since its emergence in September 2022, the BA.5 sublineage BQ.1.1 quickly spread to account for 24.2% of total COVID-19 cases by November 2022 [38, 39]. The clinical use of a bivalent vaccine targeting ancestral S protein and Omicron BA.1 S protein has been shown to result in high-level production of neutralizing antibodies when compared to boosters targeting ancestral S protein alone [40]. To overcome low vaccine effectiveness against new variants, monovalent vaccines have been replaced with bivalent vaccines for individuals aged ≥ 12 years in the U.S. and other countries since September 1st 2022 [41, 42]. Many studies have demonstrated the importance of booster doses in this context. For instance, one study showed that a booster targeting a combination of two antigens is more effective than a booster against a different variant than that targeted in the previous immunization [37]. Recent studies by Kurhade et al. have compared the effects of BA.5-targeting booster doses to parental mRNA booster doses. Following receipt of a BA.5 booster dose, protection against BA.4/5, BF.7, BA.4.6, and BA.2.75.2 is enhanced, while resistances to BQ.1.1 and XBB.1 are minimally affected [39]. WHO classified Omicron XBB.1.5 as moderate risk on 25 January 2023. Up to this time almost 54 countries have been reported this variant and most of the infection are from U.S [14]. Vaccine-elicited neutralization against these Omicron sublineages has been studied and it has been shown that individuals with the WT vaccine exhibit less neutralization against the BQ.1 sublineages and are mostly ineffective against the XBB.1 sublineages when compared to those with the bivalent (BA.5) booster shot. [43]. Furthermore, these Omicron sublineages diminish the neutralizing ability of approved monoclonal antibodies [44]. mRNA technology could speed up the preparation of the antigen against the circulating variant, and the combinations of antigens from two different variants might overcome current problems with immune escape and low vaccine efficacy. While this approach is feasible, questions remain as to which combinations would be most effective for recent or future VOCs. Many recent studies have made detailed comparisons of different variant combinations, but information on newly emerging variants is lacking. Here, we compared different bivalent combinations targeting BA.5 + WT, BA.5 + Alpha, and BA.5 + Delta to determine which was the most effective.

In this study, we perform a side-by-side comparison of monovalent and bivalent mRNA-LNP vaccine efficacies against WT, Alpha, Delta, and BA.5. We also compared the efficacies of different variant antigen combinations to protect against VOCs. The sera from mice receiving monovalent vaccines against BA.5 mRNA-LNP showed low binding affinity towards other VOCs, but these sera also showed high neutralizing activities for BA.5 sublineages BF.7 and BQ.1.1. This finding may be attributed to the fact that mutations in BA.5 separate the lineage from other VOCs; however, its sublineages remain similar to each other. These results are consistent with observations that mutations in BA.5 S protein reduce binding strength and enhance immune evasion [45, 46]. Detailed investigation of neutralizing antibody titers from immunized mouse sera revealed that bivalent vaccines are generally more effective against most relevant variants as compared with monovalent vaccines. Importantly the WT vaccine showed no neutralizing activity against BA.5 and its sublineages. In support of this idea, even booster shots of vaccines and therapeutic monoclonal antibodies designed against the WT variant showed very little protection against Omicron BA.5 in clinical trials [47, 48]. The designed bivalent vaccine induced significant neutralization activities for WT, Alpha, Delta, BA.5, and BF.7, with fairly modest activity for BQ.1.1. Meanwhile, the sera from BA.5 vaccinated mice also showed no neutralizing activity against other SARS-CoV-2 VOCs. Nevertheless, the bivalent vaccines produced significant neutralizing effects toward WT, Alpha, Delta, and BA.5. Combining BA.5 and WT (BA.5 + WT) showed a broader neutralizing response than BA.5 + Alpha and BA.5 + Delta. Thus, a bivalent vaccine could prevent recent and future breakthrough strains by ensuring the production of robust neutralizing antibody titers. Still, the exact reason for bivalent vaccine superiority remains unknown. In a recent study, a bivalent boosting group had significantly higher levels of neutralizing antibodies than a monovalent boosting group, but there was no difference in T cell response between the two groups [49]. Further studies comparing the T cell responses to bivalent vaccines and monovalent vaccines are expected to provide a more comprehensive understanding of the efficacy of these approaches. In the present study, our data suggest that BA.5 + WT combination might help to preserve the epitope repertoire against different VOCs.

## Conclusions

Mutations in S protein are always challenging because they might alter vaccine efficacy and increase virus transmissibility. Combining vaccines from two mutants is relatively uncommon, primarily due to the need for focusing on currently circulating VOCs. Here, we identified the most effective combination regimens that neutralize most SARS-CoV-2 variants. Serum from monovalent vaccine-receiving mice can bind to cross-variants, but the neutralizing activities are very low. In contrast, bivalent vaccines, particularly BA.5 + WT, are highly effective in neutralizing SARS-CoV-2 variants. Therefore, it may be possible to overcome the low vaccine efficacies against the current Omicron sublineages and future VOCs by designing a bivalent vaccine strategy. As a whole, these findings support the notion that individuals boosted with bivalent vaccines may receive better protection than those boosted with existing vaccination regimens.

## Supplementary information

**Additional file 1: Fig. S1. **Body weight was evaluated each week after immunization of mice with monovalent vaccinesor bivalent vaccine.

**Additional file 2: Fig. S2. **Graphical figure with half-maximal inhibitory concentrations (IC50) values for serum of monovalent or bivalent mRNA-vaccinated mice against SARS-CoV-2 variant pseudoviruses. Graphical data are shown as mean ± SEM. *p < 0.05, **p < 0.01, ***p < 0.001, two-tailed Student t-test.

## Data Availability

The datasets analyzed in the current study are available upon reasonable request.

## Abbreviations

COVID-19: Coronavirus disease 2019
SARS-CoV-2: Severe acute respiratory syndrome coronavirus 2
hACE2: Human angiotensin converting enzyme 2
IC_50_: Half maximal inhibitory concentration
VOCs: Variants of concern
